# The effect of serum starvation on tight junctional proteins and barrier formation in Caco-2 cells

**DOI:** 10.1016/j.bbrep.2021.101096

**Published:** 2021-08-07

**Authors:** Aisling M. Ross, Darragh R. Walsh, Rachel M. Cahalane, Lynnette Marcar, John J.E. Mulvihill

**Affiliations:** a Bioscience and Bioengineering Research (BioSciBer), Bernal Institute, University of Limerick, Ireland; b School of Engineering, University of Limerick, Ireland; c Health Research Institute (HRI), University of Limerick, Ireland; d Education and Health Sciences, University of Limerick, Ireland

**Keywords:** In vitro model, Serum-free, Transendothelial electrical resistance (TEER), Occludin, Zonula occludens-1 (ZO-1), Drug delivery

## Abstract

Assessing the ability of pharmaceutics to cross biological barriers and reach the site-of-action requires faithful representation of these barriers *in vitro*. Difficulties have arisen in replicating *in vivo* resistance *in vitro*. This paper investigated serum starvation as a method to increase Caco-2 barrier stability and resistance. The effect of serum starvation on tight junction production was examined using transwell models; specifically, transendothelial electrical resistance (TEER), and the expression and localization of tight junction proteins, occludin and zonula occludens-1 (ZO-1), were studied using western blotting and immunofluorescence. Changing cells to serum-free media 2 days post-seeding resulted in TEER readings of nearly 5000 Ω cm^2^ but the TEER rapidly declined subsequently. Meanwhile, exchanging cells to serum-free media 4–6 days post-seeding produced barriers with resistance readings between 3000 and 4000 Ω cm^2^, which could be maintained for 18 days. This corresponded to an increase in occludin levels. Serum starvation as a means of barrier formation is simple, reproducible, and cost-effective. It could feasibly be implemented in a variety of pre-clinical pharmaceutical assessments of drug permeability across various biological barriers with the view to improving the clinical translation of novel therapeutics.

## Highlights

• Serum starvation increases the intracellular resistance of Caco-2 cells.
• Max TEER values of 4783 ± 610 Ω cm^2^ were achieved in serum free conditions.
• A barrier of 3000–4000 Ω cm^2^ could be maintained for up to 18 days.
• Serum starvation leads to a significant increase in occludin expression.
• Occludin levels correlate significantly with corresponding TEER values.

## Graphical abstract

**Figure:**
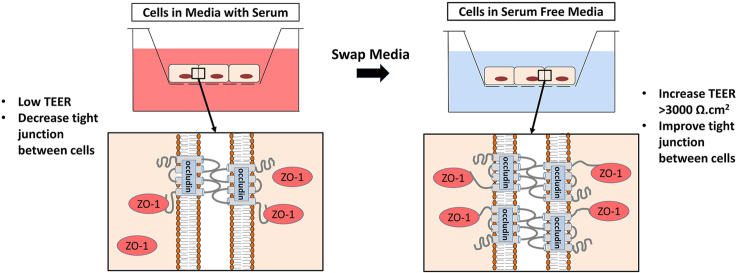

## 1 Introduction

During drug development, the solubility and permeability of drug compounds are considered major factors contributing to drug bioavailability and efficacy [1]. Processes are now being implemented to improve the water solubility of drugs [2]; however, this can pose new problems for drug permeability. For drugs to reach their intended site-of-action at therapeutic levels, they may have to cross a number of biological barriers [3] such as the gastrointestinal barrier, blood-brain barrier, blood-cerebrospinal fluid barrier, blood-ocular/blood-retinal barrier, and blood-testis/blood-epididymal barrier [4,5]. Transport of drugs across these barriers proceeds via two routes: transcellular and paracellular pathways [3]. The transcellular route is typically favored by lipophilic compounds, while hydrophilic compounds tend to cross via the paracellular route [3]. Thus, the development of more water-soluble drugs means that paracellular transport across biological barriers must be improved.

*In vitro* models are widely used in early assessments of drug permeation in an effort to reduce reliance on animal models for pre-clinical testing [6]. Here, paracellular transport is modulated by tight junctions [3] which prevent passive diffusion of molecules between adjacent barrier cells [7]. Transendothelial electrical resistance (TEER) is commonly employed to assess the resistance of these models to paracellular diffusion. The accuracy of these models relies on faithful representation of tight junctional complexes. Often, these models underproduce tight junction proteins, resulting in more permeable barriers than their *in vivo* counterparts. For example, in the context of the blood-brain barrier (BBB), TEER values *in vivo* have been measured between 2000 and 6000 Ω cm^2^[8,9]. Although, based on solute permeation coefficients, it has also been calculated to be as high as 8000 Ω cm^2^[10]. However, there is a consensus in the field that TEER values above 150 Ω cm^2^ are considered to have acceptable resistance to conduct *in vitro* permeability studies [11,12].

Estimating the permeability of drugs using *in vitro* barriers with low resistance may not be effective in predicting *in vivo* bioavailability. Attempts to improve TEER values in models include co-culturing [[13], [14], [15], [16], [17], [18], [19], [20], [21]], treatment with extracellular matrix components [[20], [21], [22], [23]] and application of fluid flow [20,21,24,25]. However, these methods are costly and labor intensive, and still may not recapitulate *in vivo* barrier resistance.

One method used to alter the expression of tight junction proteins *in vitro* is serum starvation. The presence of serum in barrier cultures has been shown to prevent tight junction formation thereby reducing TEER values and increasing paracellular transport [26]. In this paper, we investigated the effect of serum-free media on the formation of restrictive monolayers in Caco-2 cells as a simple model for *in vitro* barrier formation. Caco-2 cells are gastrointestinal epithelialia established from a human colorectal adenocarcinoma. These cells have been used extensively for the last 40 years to study polarized barriers and drug permeability [27]. Given the extensive research conducted using these cells, they are a useful model to study conditions affecting barrier permeability. We hypothesized that a period of culture in serum containing media would allow time for monolayer formation. Following this with a period of culture in serum-free media would result in differentiation of the cells, thereby improving tight junction formaton and increasing barrier resistance. In doing so, we explored the effect of serum-containing and serum-free culture conditions on the expression and localization of extracellular and intracellular tight junction protein, occludin and zonula occludens-1 (ZO-1), respectively. The formation of simple, reproducible, and cost-effective models of biological barriers will improve the accuracy of pre-clinical *in vitro* evaluation of medications [28].

## 2 Materials and methods

### 2.1 Cell culture

Caco-2 cells (Sigma Aldrich 86010202) were maintained in complete culture medium (CCM) [DMEM (Sigma Aldrich D6546) with 10 % FBS (Sigma Aldrich F7524), 1 % non-essential amino acids (Sigma Aldrich M7145), 2 mM l-glutamine (Sigma Aldrich G7513), and 100 units/mL penicillin and 100 μg/mL streptomycin (Sigma Aldrich P4333)] as described by Lea (2015) [27].

### 2.2 Transwell model

The apical side of a 0.4 μm pore, polycarbonate 12-well transwell (Corning 3401) was seeded with Caco-2 cell suspension (passage 4–6) (1 × 10^5^ cells/transwell, 8.9 × 10^4^ cells/cm^2^). After a period of culture in CCM, cells were transferred to serum-free (SF) culture medium [DMEM supplemented with 1 % non-essential amino acids, 2 mM l-glutamine, 100 units/mL penicillin and 100 μg/mL streptomycin]. To determine the optimal timepoint for transfer to SF culture medium, in different transwells CCM was exchanged for SF on a day between 0 and 20 days in culture. Cells were all maintained in culture until Day 40 (where Day 0 is the day of seeding).

### 2.3 Transendothelial electrical resistance

Every two days TEER of the cell barrier was measured using the EVOM2 (World-Precision Instruments, United Kingdom) as described by Yeste *et al.* (2018) [29]. The resistance of a blank well (membrane with no cells) was recorded as a background reading and subtracted from the measured resistance values. This value was multiplied by the membrane surface area (1.12 cm^2^) to calculate the TEER. Three measurements were acquired for each well. Conditions were tested in triplicate across three separate experiments. TEER results are presented as the mean ± standard deviation.

### 2.4 Immunoblotting

Immunoblotting was carried out similar to the method described in Walsh *et al.* (2018) [30]. For immunoblotting experiments, Caco-2 cells (passage 5–8) were seeded in 6-well plates (Corning 3516) at 8.9 × 10^4^ cells/cm^2^. Following culture, cells were collected and lysed on ice for 30 min [Note: SFD0 samples (cells exchanged to SF media on Day 0 and collected the same day) were collected 2 h post-seeding]. Protein content was quantified using a bicinchonic acid assay (Fisher Scientific 23225).

Samples containing 15 μg of protein were prepared with 1x LDS sample buffer (Invitrogen B0007) and 2.5 % β-mercaptoethanol and heated to 95 °C for 5 min. Samples were run on 4–12 % Bis-Tris gels (Invitrogen NW04125BOX) using 1x MOPS running buffer (Invitrogen B0001). Gels were transferred to nitrocellulose membranes (GE Healthcare Amershem Protran 10600003). For GAPDH and occludin, gels were transferred at 100 V for 1 h at 4 °C in transfer buffer. For ZO-1, gels were transferred in transfer buffer containing 0.1 % w/v SDS for 1.5 h at 100 V at 4 °C. Membranes were blocked for 1 h at room temperature (RT) in 5 % skim milk in tris-buffer saline-0.1 % tween 20 and probed overnight at 4 °C with primary antibody (Supplementary Material [SM]-Table S1). The membranes were incubated for 1 h at RT with secondary antibody (SM-Table S1). Membranes were imaged using the LI-COR Odyssey. Triplicate experiments were conducted.

Densitometry was conducted using the LI-COR Image Studio Lite (version 5.2). The intensity of ZO-1 and occludin were normalized against GAPDH. All intensity values were expressed relative to the SFD0 sample. The mean normalized relative intensity was calculated across triplicate experiments.

### 2.5 Immunofluorescence

Caco-2 cells (passage 5–8) were seeded in 96-well plate (Corning 3599) at a concentration of 8.9 × 10^4^ cells/cm^2^. After culturing, immunofluorescene was carried our using a similar technique to that employed by De Benedictis et al. (2021) [31]. Briefly, cells were fixed for 15 min in a 4 % formaldehyde (Note: SFD0 samples were fixed 2 h post-seeding). Cells were permeabilised for 15 min at RT in 0.5 % Triton X-100 in PBS and blocked for 1 h at RT with 1x PBS containing 10 % FBS, 0.005 % Triton X-100. Cells were probed for occludin or ZO-1 (SM-Table S1) overnight at 4 °C. Cells were incubated with secondary antibody (SM-Table S1) for 1 h at RT followed by a 10 min incubation with 0.1 μg/mL DAPI (Sigma Aldrich D9542). Cells were imaged using the ImageXpress Microconfocal High-Content Imaging System (Molecular Devices). Experimental conditions were examined in triplicate across three separate experiments. 9 images were acquired per well. The images were analyzed utilizing CellProfiler (version 3.1.8) [32]. Image analysis protocol is detailed in SM-Section 4.1.

### 2.6 Statistical analysis

Statistical analysis was carried out using Graphpad Prism 6 (version 8.3.1). For immunoblot analysis, normalized protein levels are expressed as mean normalized relative intensity ± standard error about the mean (SEM). Mean protein levels were compared using one-way analysis of variance (ANOVA) at a 95 % confidence interval with Sidak's multiple comparisons test. Correlations between protein levels and TEER values were analyzed using both Pearson's and Spearman's correlations. Spearman's correlation was used to compare the protein levels and corresponding TEER values at the same time point. Meanwhile, Pearson's correlation was used to compare proteins levels at the time the cells were transferred to SF media and the maximum TEER values.

For immunofluorescence, the normalized protein intensity for each image was compared to others from the same well using the ROUT outliers test (Q = 1) and outliers were excluded. Mean intensities for replicate wells were similarly compared. All intensity values were expressed as mean ± SEM. Mean intensities were compared using one-way ANOVA using a 95 % confidence interval with Sidak's multiple comparisons test.

## 3 Results

A number of methods were utilized to assess the role of serum-free (SF) media on tight junctions formation in Caco-2 monolayers. In addition to using TEER to determine monolayer resistance, the impact of serum on tight junction protein expression and sub-cellular localization was investigated.

### 3.1 Changes in TEER in response to serum-containing and serum-free media

**Fig. 1:**
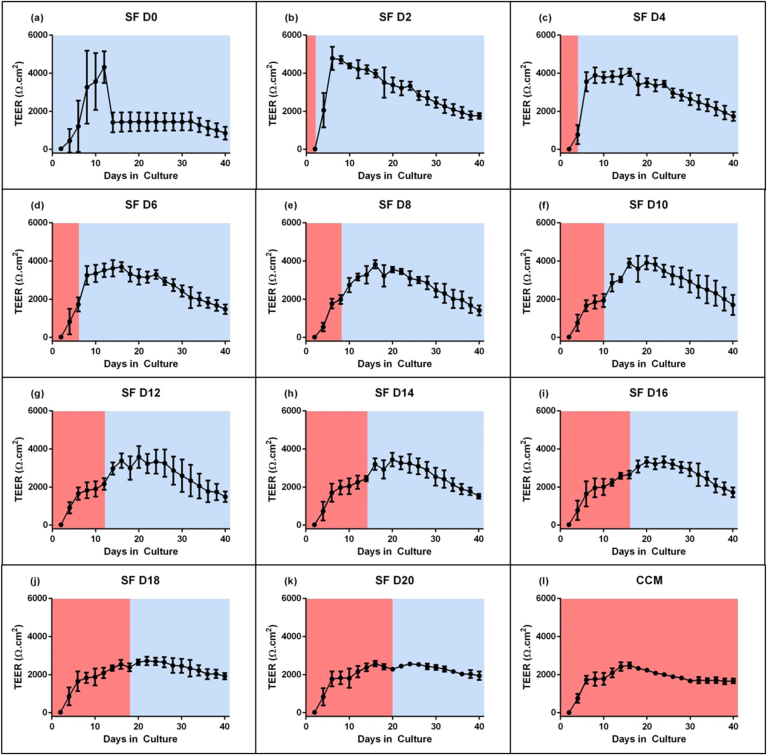
Transendothelial electrical resistance (TEER) of cell barriers was measured every 2 days using the EVOM2. Complete culture media (CCM) was exchanged for serum-free (SF) media on different days (e.g. SFD4 denotes that media was exchanged to serum-free media following 4 days in CCM). The period of the graphs relating to culture in CCM is highlighted in red and culture in SF media is highlighted in blue. Data points represent the mean of triplicate measurements across triplicate wells from triplicate experiments (n = 27). Error bars denote standard deviation. (For interpretation of the references to color in this figure legend, the reader is referred to the Web version of this article.)

Culture in SF media has been shown to increase barrier resistance to paracellular transport *in vitro* [26,33]. We sought to investigate this in the context of Caco-2 barriers. To evaluate the formation of a restrictive barrier, TEER was measured every 2 days for 40 days (Fig. 1).

Based on this assessment, switching to SF media early in the culture period (SFD0, SFD2) resulted in the highest maximum TEER (Fig. 1a and b; SM-Table S2). However, the measurements showed significant variability and values subsequently decreased rapidly.

Instead, cells exchanged to SF media on day 4–6 (Fig. 1c and d) achieved TEER measurements between 3000 and 4000 Ω cm^2^. These measurements showed lower standard deviations and the barriers remained above 3000 Ω cm^2^ for over 2 weeks.

Meanwhile, longer periods of culture in CCM prior to exchanging for SF media resulted in lower and more variable TEER values (Fig. 1e–k). When exchanging to SF media on D20, TEER values are not higher than wells maintained in CCM for the duration (Fig. 1k-l).

### 3.2 Occludin and ZO-1 expression and barrier formation

**Fig. 2:**
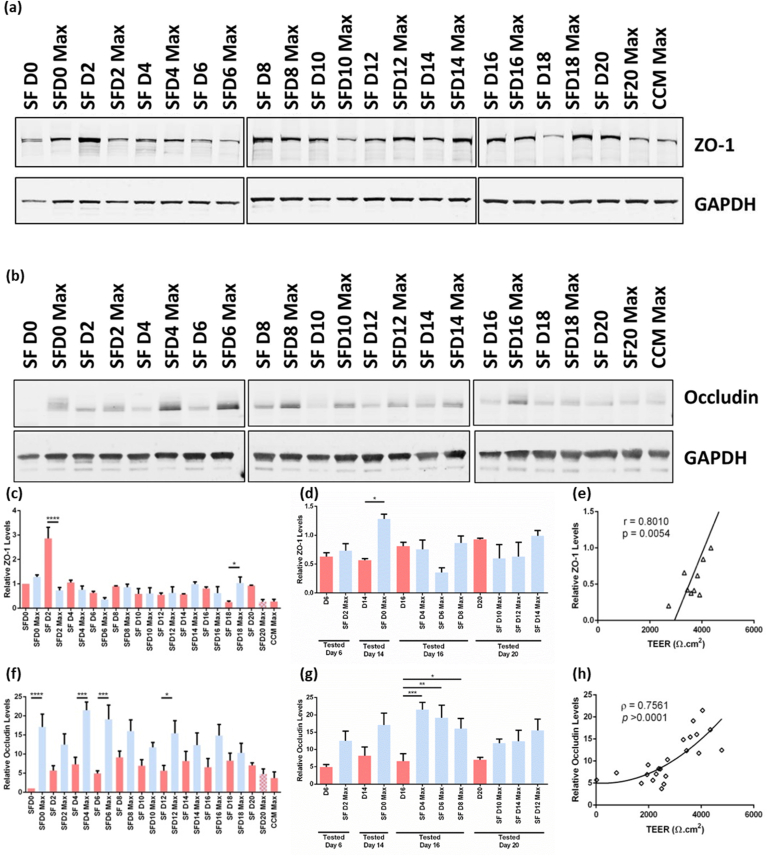
Immunoblot analysis of ZO-1 and occludin levels. Representative immunoblot showing **(a)** ZO-1 and **(b)** occludin with GAPDH as an internal control. **(c)** Relative ZO-1 levels (relative to SFD0; n = 3). **(d)** Relative ZO-1 levels grouped according to days in culture at the time of analysis. **(e)** Correlation between relative ZO-1 levels at the time the cultures are transferred to SF media and maximum TEER. **(f)** Relative occludin levels (relative to SFD0; n = 3). **(g)** Relative occludin levels grouped according to days in culture at the time of analysis. **(h)** Correlation between relative occludin levels and TEER. SF=Serum-free, D = day; SFD0 = cells collected on D0; SFD0-Max = cells exchanged to serum-free media on day 0, collected on the day these cultures achieve maximum resistance. Bars in red represent cultures in CCM; bars in blue represent protein levels at maximum TEER values after culture in SF media, with the exception of SFD20-Max (blue and red hatched bar) which reached maximum TEER in CCM before transfer to SF media. Bars represent the mean (n = 3) and error bars represent SEM. * = 0.05 > *p* > 0.01; ** = 0.01 > *p* > 0.001; *** = 0.001 > *p* > 0.0001; **** = 0.0001 > *p*. (For interpretation of the references to color in this figure legend, the reader is referred to the Web version of this article.)

To investigate the reason for these changes in TEER, we conducted immunoblot analysis of the levels of tight junction proteins, occludin and ZO-1 (Fig. 2) (raw immunoblots in SM-Fig. S1). We analyzed protein expression levels every 2 days from D0-D20 and at the point that the respective cultures achieved maximum TEER. In this way, we could analyze:

1. Changes in protein levels over 20 days in CCM i.e. SFD0, SFD2, etc.,
2. Protein levels before switching to SF media compared to when maximum TEER was achieved (e.g. SFD0 vs SFD0-Max),
3. The relationship between protein levels and TEER.

**Fig. 3:**
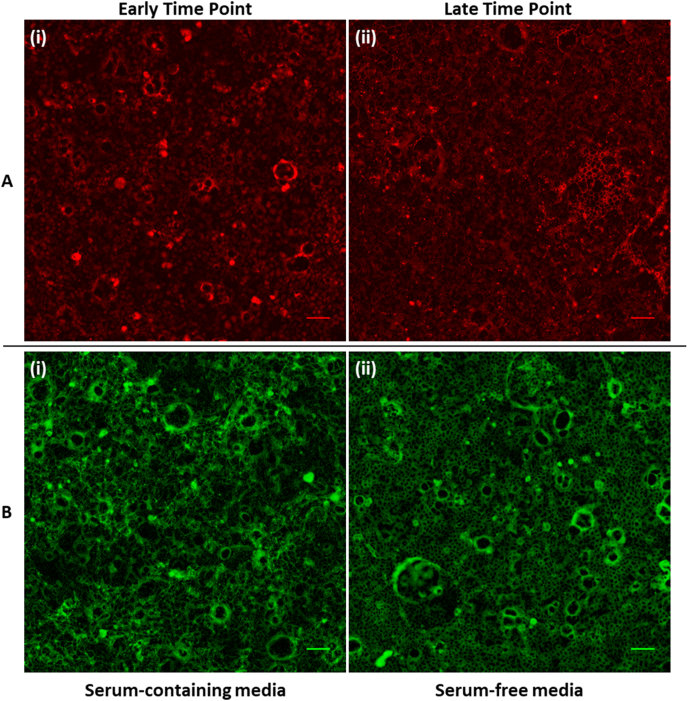
Representative immunofluorescence images of ZO-1 and occludin. Representative images of **(A)** ZO-1 (red) at **(i)** an early time point (SFD4) and **(ii)** a late time point (SFD18-Max), and of **(B)** occludin (green) maintained in **(i)** serum containing media (CCM-Max) and **(ii)** transferred to SF media (SFD2-Max). Scale bar = 100 μm. SF=Serum-free, D = day; D0 = cells changed to SF media on Day 0; SFD0 = cells exchanged to SF media on day 0, fixed that day i.e. on day 0; SFD0-Max = cells exchanged to serum-free media on day 0, fixed on the day these cultures achieve maximum resistance. (For interpretation of the references to color in this figure legend, the reader is referred to the Web version of this article.)

Immunoblotting showed that ZO-1 levels increase early in the culture period with maximum ZO-1 expression occurring on D2 (Fig. 2a&c; SFD2). After this, ZO-1 levels decrease and remain relatively stable with no significant variation in the quantity of ZO-1 (Fig. 2c; red bars). The transfer of cells to SF media resulted in no significant alteration of ZO-1 levels, with the exception of those transferred to SF media on D2 (SFD2 vs SFD2-Max); here there was a significant decrease in ZO-1 levels between levels on day 2 (SFD2) and the day this culture reached maximum TEER (SFD2-Max) (Fig. 2c).

We compared the expression of ZO-1 in cultures of CCM and SF media, analyzed after the same total culture time (Fig. 2d). This showed that there was no significant difference between different culture conditions, analyzed after the same length of culture. Thus, we concluded that SF media does not appear to effect ZO-1 expression.

Conversely, occludin levels are relatively low in CCM. A significant difference between protein levels on the day the culture is exchanged to SF media and the point the culture reach maximum TEER can be seen (Fig. 2b&f).

To investigate whether this resulted from a longer culture period or a response to SF conditions, we examined the expression of occludin between cultures in CCM and SF media, analyzed after the same length of time in culture (Fig. 2g). SFD16 was analyzed following 16 days culture in CCM, while SFD4-Max, SFD6-Max, and SFD8-Max had been cultured in SF media for 12, 10 and 8 days, respectively, for a total 16 days in culture; therefore, these experimental conditions had been in culture for the same length of time but in different conditions. This showed that there was a significant difference between SFD16 and SFD4-Max (*p* = 0.0003), SFD16 and SFD6-Max (*p* = 0.0027), and SFD16 and SFD8-Max (*p* = 0.0460), with the largest difference corresponding to the longest period in SF media (SFD16 vs SFD4-Max). This indicated that greater levels of occludin expression resulted from SF conditions not increased culturing time.

To examine the relationship between tight junction expression and barrier resistance, we investigated the correlation between relative protein levels and TEER. Occludin levels significantly correlated to TEER (ρ = 0.7561, *p* > 0.0001) (Fig. 2h). However, ZO-1 levels showed no significant correlation (ρ = 0.04122, *p* = 0.8555; SM-Fig. S2a). Interestingly, since ZO-1 was largely expressed in CCM conditions, we investigated the correlation between the ZO-1 levels at the time of exchange to SF media and the maximum TEER values. This showed that there was a significant correlation between the ZO-1 levels when the cultures were transferred to SF media and the maximum TEER achieved (r = 0.8010, *p* = 0.0054) (Fig. 2e). This was not seen for occludin (SM-Fig. S2b).

### 3.3 Localization of occludin and ZO-1 in barrier resistance

Increased tight junction expression alone is not sufficient for barrier resistance; localization of these proteins to the cell membrane is necessary. When protein localization is considered, it is evident that in cultures maintained in serum-containing media for extended periods (over 10 days), occludin localization becomes more diffuse or punctate (Fig. 3Bi). The optimum localization in serum-containing media is seen in the early cultures (SM-Fig. S5). Meanwhile, samples that had been cultured SF media show improved localization of occludin to the cell membrane, even after 10 days (e.g. SFD2-Max (Fig. 3Bii)).

Conversely, for ZO-1, as with immunoblot analysis, protein localization does not appear to be serum-dependent. There is no distinct difference between cell membrane localization in CCM compared to SF media (SM-Fig. S4). Instead, it appears that the early cultures (SFD0, SFD2, SFD4) (Fig. 3Ai) show more diffuse staining with localization becoming more pronounced in the later days (SFD14-SFD20 and SFD10-Max to SFD18-Max) irrespective of culture media (Fig. 3Aii). Staining can still be seen within the cytoplasm but we attribute this to continuous production of the protein.

**Fig. 4:**
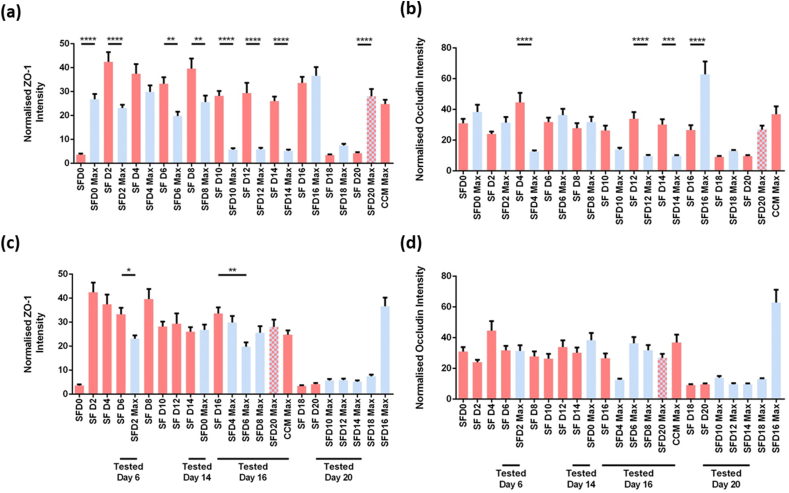
Immunofluorescence analysis of ZO-1 and occludin levels. **(a)** Normalized ZO-1 intensity (n = 9). **(b)** ZO-1 intensity organized according to days in culture at the time of analysis. **(c)** Normalized occludin intensity (n = 9). **(d)** Occludin intensity organized according to days in culture at the time of analysis. SF=Serum-free, D = day; SFD0 = cells collected on D0; SFD0-Max = cells exchanged to serum-free media on day 0, collected on the day these cultures achieve maximum resistance. Bars in red represent cultures in CCM at the point that the cultures would be transferred to SF media; bars in blue represent protein levels at maximum TEER values after cultured in SF media, with the exception of SFD20-Max (blue and red hatched bar) which reaches maximum TEER in CCM before transferring to SF media, Bars represent the mean (n = 3) and error bars represent SEM. * = 0.05 > *p* > 0.01; ** = 0.01 > *p* > 0.001; *** = 0.001 > *p* > 0.0001; **** = 0.0001 > *p*. (For interpretation of the references to color in this figure legend, the reader is referred to the Web version of this article.)

The relative signal intensity of each protein (normalized by number of cells) was compared (Fig. 4b). Occludin levels show different expression patterns to those seen in the immunoblot analysis (Fig. 4c). The mismatch between immunoblot and immunofluorescence results may be due to epitope accessibility. In the serum-containing cultures, occludin is more diffuse and, thus, the occludin epitope is more accessible for antibody binding. Meanwhile, in the serum-free conditions, occludin localization to the cell membrane results in compact, complex protein interactions which may shield extracellular epitopes from antibody binding [34]. Hence, in these conditions, total protein could be underestimated.

## 4 Discussion

Serum starvation is believed to improve barrier formation by reducing proliferation and promoting cell differentiation [33,35,36] thereby increasing barrier formation and reducing permeability [33]. Factors derived from serum are reported to destabilize already formed tight junctions, thereby increasing model permeability [26,37]. We postulated that a period of culture in serum-containing media is initially required to promote cell proliferation and initial barrier formation. This would then be followed by a period in SF media to promote increased cell differentiation, tight junction expression and localization. Extended time in serum-containing media could result in de-differentiation of the cells while premature transfer to SF condition could lead to poor monolayer formation. Therefore, we considered that the balance between culture in serum-containing and serum-free media would affect the quantity and quality of the tight junctions formed.

The results presented here show that a period of 4 days in CCM followed by 6–12 days in SF media resulted in the most stable and restrictive barrier. We believe that these conditions facilitated complete monolayer formation (immunofluorescence) and optimum expression of occludin and ZO-1 (immunoblotting).

Occludin is a critical transmembrane tight junction protein [38,39], forming homocomplexes with neighboring cells [40]. This is believed to be responsible for establishing a seal at the site of junctional complexes [3]. ZO-1, unlike occludin, is an intracellular protein involved in anchoring occludin and other tight junction proteins to the cytoskeleton, providing stability to junctional complexes [39,41]. As expected, occludin levels were found to correlate significantly with TEER and corresponded to the conditions that resulted in the most restrictive and stable barrier formation. This indicates that occludin levels not only confer increased resistance but also lead to increased barrier stability, likely resulting from improved extracellular complexes.

It is possible that the increased occludin levels are the result of activation of a p38 MAPK pathway. p38 mitogen-activated protein kinases (MAPKs) are MAPK isoforms that are involved in cell differentiation [42] and stress response [43]. It has been shown previously that endostatin can act in a p38 MAPK and ERK1/2 (extracellular signal-regulated kinase) dependent manner to upregulate occludin expression and hence decrease barrier permeability in a blood-retinal barrier model [42,44]. Further, it has also been shown that serum starvation can induce phosphorylation of p38 [45,46] in addition to activation of ERK-1 and ERK-2 [47]. Taken together, these results may suggest that serum-starvation here induced an increase in occludin expression and corresponding decrease in permeability through activation of a p38 MAPK and ERK1/2 pathway. However, further testing would be required to elucidate the involvement of this pathway.

We expected to see a similar trend for ZO-1 expression. However, instead we found that ZO-1 levels did not change significantly following transfer to SF media. The highest levels of ZO-1 occurred on D0 and D2. It is worth noting that the variability seen in the ZO-1 samples in the initial days of culture is likely the result of ZO-1 present in the cells at the time the experiment was established. For occludin, as the protein contains an extracellular portion, during sub-culturing the protein will likely be fragmented by trypsin [48]. It will then be synthesized *de novo* following experimental set-up. This is supported by the low levels of occludin at D0 which steadily increased throughout the experiment. Conversely, ZO-1 is an intracellular protein and would not be digested by trypsin. Therefore, D0 levels are likely to be related to the ZO-1 levels within the cells at the time of experimental set-up. This resulted in higher initial ZO-1 levels than expected and greater inter-experiment variability. After this, ZO-1 levels decrease and remain relatively stable regardless of SF or CCM culture conditions. This would indicate that ZO-1, unlike occludin, is not stimulated by SF conditions but it is produced consistently in CCM or SF media.

ZO-1 plays a role in the organization of the tight junctions, trafficking occludin to the membrane in the early days of barrier formation [41]. Previously, when cells are transfected with occludin, the protein cannot be trafficked to the cell membrane in the absence of ZO-1 [49,50]. Our results support this concept; while ZO-1 levels do not directly correlate to TEER, the presence of increased levels of ZO-1 at the point the cultures are switched to SF media could be involved in trafficking the newly formed occludin to the cell membrane. Thus, ZO-1 levels are likely to correspond indirectly to barrier resistance. Meanwhile, increased occludin correlated directly to improved barrier resistance and stability.

In addition to the use of *in vitro* models, such as the model described here, to directly assess drug permeability across biological barrier, such models could also be used in conjunction with computational models of barrier permeability [51]. The use of *in silico* models has increased in the last 20 years with continuous improvements on modeling capabilities and machine learning [52,53]. Computational models utilize *in vivo* and *in vitro* data to inform modeling and improve prediction capabilities [[53], [54], [55]]. Better *in vitro* models can provide a better understanding of the mechanisms by which drugs permeate across biological barriers [54]. Together improved *in vitro* and *in silico* models can work synergistically to reduce our reliance on animal models in the drug development process.

## 5 Conclusions

Utilizing unrealistic, “leaky” *in vitro* barrier models will over-estimate pharmaceutical paracellular permeation across biological barriers, ultimately wasting time and money. Here, we investigated the effects of serum starvation on Caco-2 cell barrier formation. We found that exchanging cells from serum-containing media to serum-free media on day 4 resulted in TEER values of 3000–4000 Ω cm^2^ which could be maintained for >2 weeks. We have attributed this increase in resistance to higher levels of occludin caused by serum starvation. Newly formed occludin is likely trafficked to the cell membrane by ZO-1; ZO-1 levels seem to indirectly correlate with maximum TEER. However, ZO-1 does not appear to be affected by serum levels, directly. To conclude, serum starvation could be a simple, rapid, reproducible, and cost-effective method to prepare biological barrier models. Such models could be easily implemented in preliminary pharmaceutical assessments to evaluate the ability of novel therapeutics to reach their site-of-action at clinically significant levels.

## Funding

The University of Limerick, Faculty of Science and Engineering Postgraduate Scholarship, 2016.

## Author Contributions

**Aisling M.Ross:** Conceptualization, Methodology, Formal Analysis, Investigation, Data Curation, Resources, Writing - Original Draft, Visualization.

**Rachel M. Cahalane:** Methodology, Investigation, Resources, Writing – Review and Editing.

**Darragh R.Walsh:** Methodology, Investigation, Formal Analysis, Software, Resources, Writing – Review and Editing.

**Lynnette Marcar:** Conceptualization, Methodology, Formal analysis, Data Curation, Visualization, Project Administration, Writing – Review and Editing.

**John J.E.Mulvihill:** Conceptualization, Resources, Writing – Review and Editing, Supervision, Project Administration, Funding Acquisition.

## Declaration of competing interest

None.
