# Supplementary material for: The effect of serum starvation on tight junctional proteins and barrier formation in Caco-2 cells
Source: Biochem Biophys Rep. 2021 Aug 7;27:101096. doi: 10.1016/j.bbrep.2021.101096 (PMC8358646; doi:10.1016/j.bbrep.2021.101096)
Supplement: Multimedia component 1 [file mmc1.pdf]

Supplementary Material

1 Antibodies

Table S1: Summary of the antibodies used for immunoblotting and immunofluorescence.

	**Primary Antibody**			**Secondary Antibody - Immunoblotting**			**Secondary Antibody - Immunofluorescence**		
	**Conc.**	**Supplier**	**Product Code**	**Conc.**	**Supplier**	**Product Code**	**Conc.**	**Supplier**	**Product Code**
**GAPDH**	1:5,000	Biosciences (Invitrogen)	PA1-987	1:10,000	Biosciences (Invitrogen)	A11375	N/A	N/A	N/A
**Occludin**	1:500	Sigma Aldrich	SAB3500301	1:10,000	Biosciences (Invitrogen)	A21076	1:2,000	Sigma Aldrich	SAB4600069
**ZO-1**	1:500	Biosciences (Invitrogen)	61-7300	1:10,000	Biosciences (Invitrogen)	A21076	1:2,000	Sigma Aldrich	SAB4600069

2 TEER Readings

Table S2**:** Summary of sample collection. Sample collection time-points, sample culture media at the time of collection and the corresponding TEER values at the time of collection. CCM = Complete culture media, SF = Serum free, D = day; SFD4 = cells exchanged to SF media of D4, after 4 days in CCM; SFD0 Max = cells exchanged to serum free media on day 0, collected on the day these cultures achieve maximum resistance.

**Sample**	**Day Sample Collected**	**Media at time of collection**	**TEER on day of collection (mean ± SD) (Ω.cm^2)**
**SF D0**	Day 0	CCM	Not Read
**SFD0 Max**	Day 14	SF	4344 ± 214
**SF D2**	Day 2	CCM	19 ± 15
**SFD2 Max**	Day 6	SF	4783 ± 610
**SF D4**	Day 4	CCM	775 ± 503
**SFD4 Max**	Day 16	SF	4047 ± 191
**SF D6**	Day 6	CCM	1723 ± 367
**SFD6 Max**	Day 16	SF	3698 ± 246
**SF D8**	Day 8	CCM	1982 ± 233
**SFD8 Max**	Day 16	SF	3828 ± 214
**SF D10**	Day 10	CCM	1937 ± 346
**SFD10 Max**	Day 20	SF	3912 ± 352
**SF D12**	Day 12	CCM	2162 ± 300
**SFD12 Max**	Day 20	SF	3581 ± 575
**SF D14**	Day 14	CCM	2443 ± 137
**SFD14 Max**	Day 20	SF	3443 ± 352
**SF D16**	Day 16	CCM	2651 ± 204
**SFD16 Max**	Day 24	SF	3323 ± 286
**SF D18**	Day 18	CCM	2385 ± 207
**SFD18 Max**	Day 22	SF	2720 ± 219
**SF D20**	Day 20	CCM	2287 ± 24
**SFD20 Max**	Day 16	CCM[a]	2580 ± 145
**CCM Max**	Day 16	CCM	2491 ± 154

[a]Sample D20 Max reached maximum TEER prior to exchanging CCM for SF Media; switching to SF media did not result in any increase in TEER.

3 Immunoblotting

3.1 Raw Immunoblots

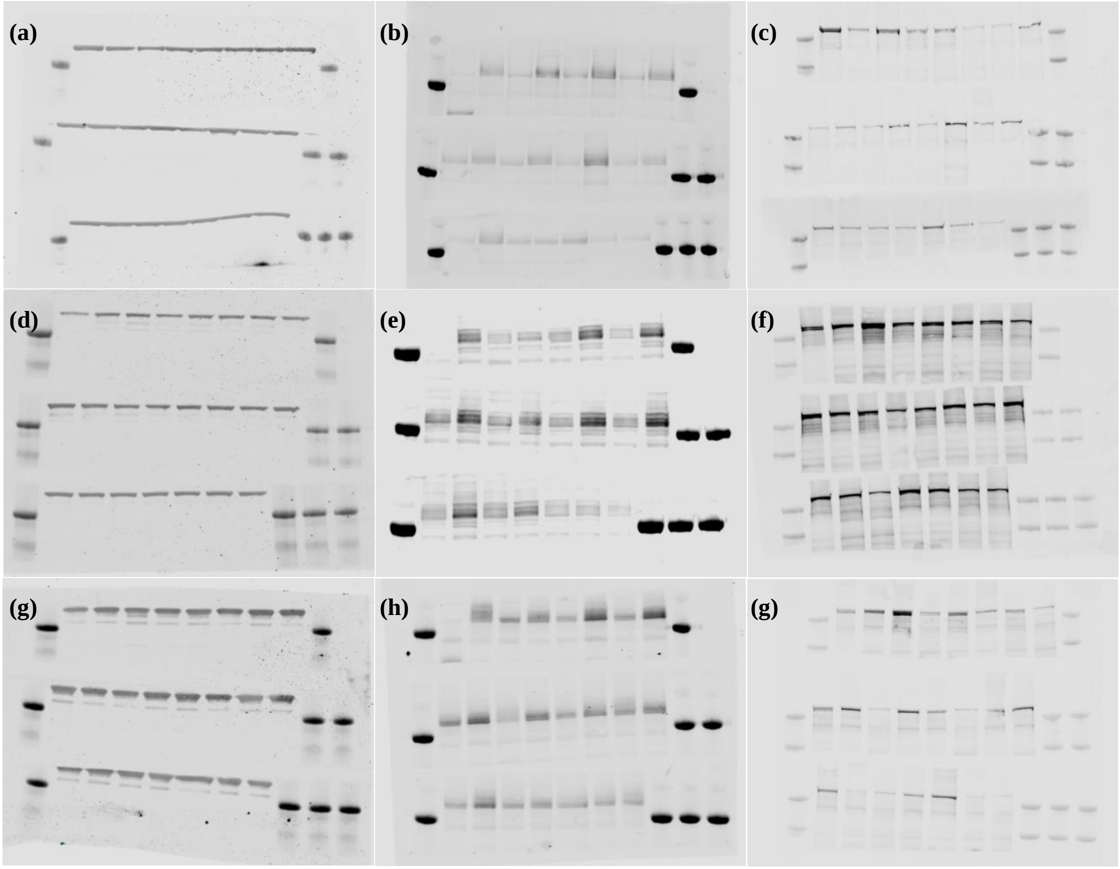

Figure S1: Raw western blot images (a) Experiment 1: GAPDH, (b) Experiment 1: Occludin, (c) Experiment 1: ZO-1, (d) Experiment 2: GAPDH, (e) Experiment 2: Occludin, (f) Experiment 2: ZO-1, (g) Experiment 3: GAPDH, (h) Experiment 3: Occludin, (i) Experiment 3: ZO-1. For all images lanes are as follows: Top Blot (L-R) Ladder, SF D0, SF D0 Max, SF D2, SF D2 Max, SF D4, SF D4 Max, SF D6, SF D6 Max, Ladder; Middle Blot (L-R) Ladder, SF D8, SF D8 Max, SF D10, SF D10 Max, SF D12, SF D12 Max, SF D14, SF D14 Max, Ladder, Ladder; Bottom Blot (L-R) Ladder, SF D16, SF D16 Max, SF D18, SF D18 Max, SF D20, SF D20 Max, CCM Max, Ladder, Ladder, Ladder. SF = Serum free; D = day; D0 = cells collected on D0; SFD0 Max = cells exchanged to SF on D0, collected on the day cultures achieve maximum resistance; CCM = complete culture media.

3.2 Correlations between Protein Levels and TEER

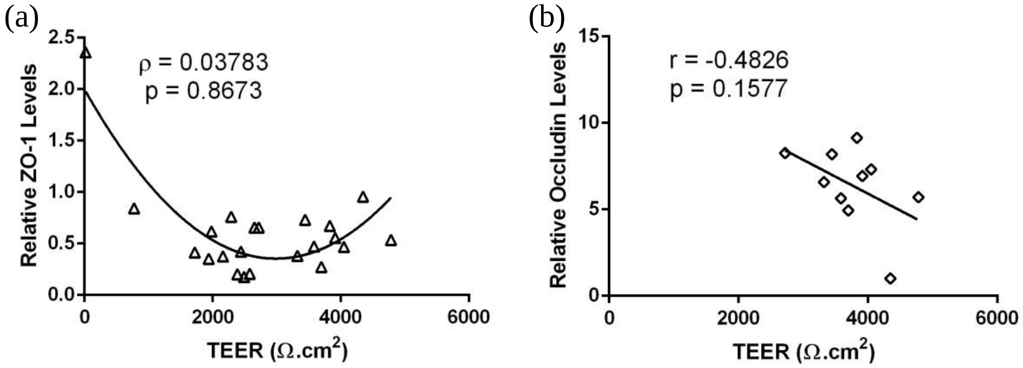

Figure S2: (a) Correlation between relative ZO-1 levels and TEER values. (b) Correlation between relative Occludin levels at the time the cultures are transferred to SF media and maximum TEER values. SF = Serum free, TEER = Transendothelial electrical resistance.

4 Immunofluorescence

4.1 Image Acquisition

All images were acquired using automated confocal imaging on the Molecular Devices ImageXpress High-Content Microconfocal. Focusing and exposure optimization were carried out on a small subset of wells across the range of the plate. The sites used to optimize the imaging conditions were not acquired for image analysis to avoid photobleaching which could affect the images used for quantitation. Automated imaging uses rapid, plate bottom- and well bottom-based autofocusing to ensure each individual site is in focus before acquiring the images. Any potential bleaching caused by autofocusing would be negligible as the DAPI channel is used and it is uniformly applied to all wells and sites. All images were acquired at the same magnification, exposure, and laser intensity.

4.2 Image Analysis using CellProfiler

The acquired immunofluorescence images were analyzed using the open source CellProfiler software (Version 3.1.8, Graphpad, USA). First, the number of cells in each image was quantified by counting the number of DAPI-stained nuclei. To do this, the DAPI images were first corrected to adjust for potential inhomogeneous sample illumination. The illumination function for the DAPI-stained images was calculated using the ‘background’ method and the illumination-corrected images were calculated by subtracting the illumination function from the original images (see Figure S3). Next, the ‘IdentifyPrimaryObjects’ module was utilized to segment and quantify the number of nuclei within the illumination-corrected DAPI image. Illumination correction was also conducted on the occludin and ZO-1 Images. One background image was calculated for the occludin-stained cell images per experiment and another for the ZO-1-stained images. Following illumination correction, the total fluorescence intensity was quantified by measuring the sum of all pixel intensity values within each image. To normalize the total intensity of fluorescent signal by the number of cells present, the total image intensity was divided by the number of cells identified in the corresponding DAPI image.

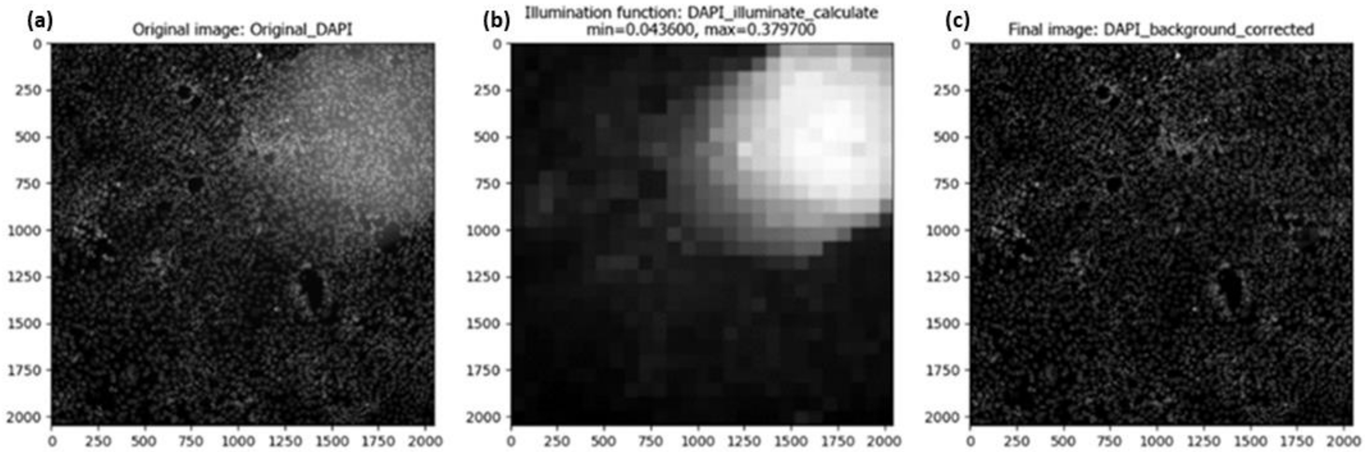

Figure S3: CellProfiler illumination correction workflow. (a) The original image contains uneven illumination-related noise as evidenced by the white section at the top right of the image. (b) The illumination function calculated using the 'background' method. (c) The illumination-corrected image is not subject to the uneven illumination and can thus be quantitatively analyzed.

4.3 ZO-1

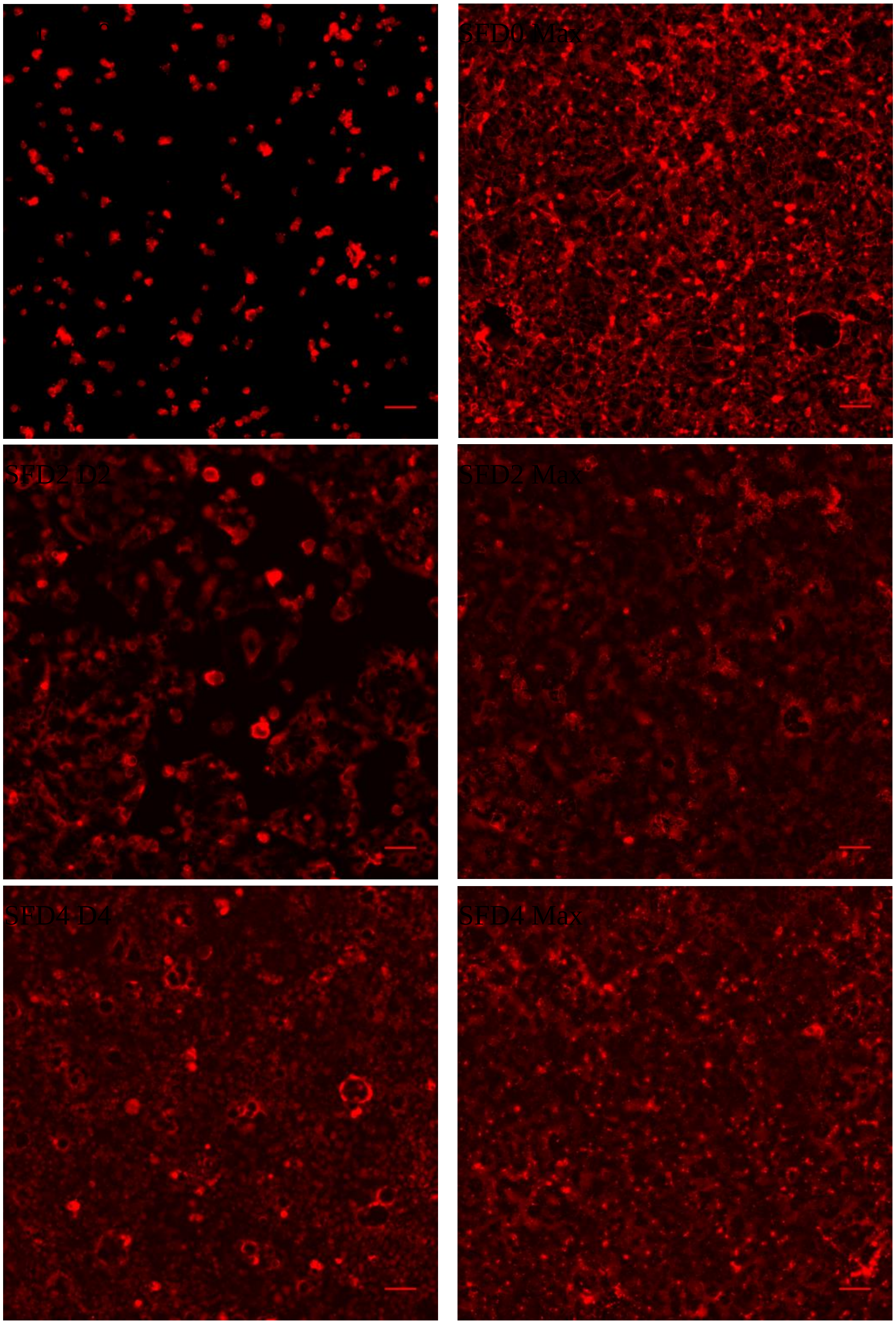

SFD0 Max
SFD2 D2
SFD2 Max
SFD4 D4
SFD4 Max

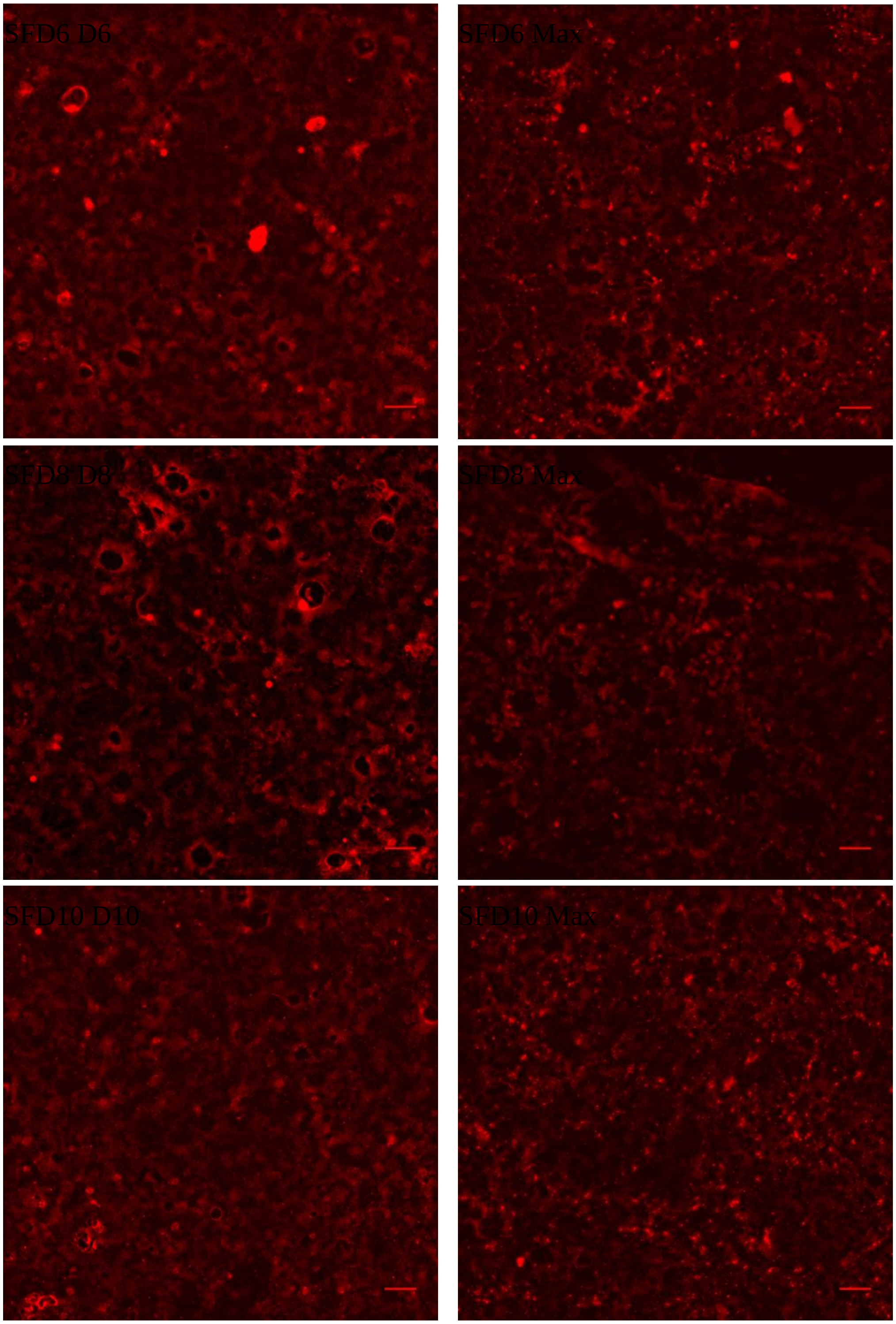
SFD6 D6
SFD6 Max
SFD8 D8
SFD8 Max
SFD10 D10
SFD10 Max

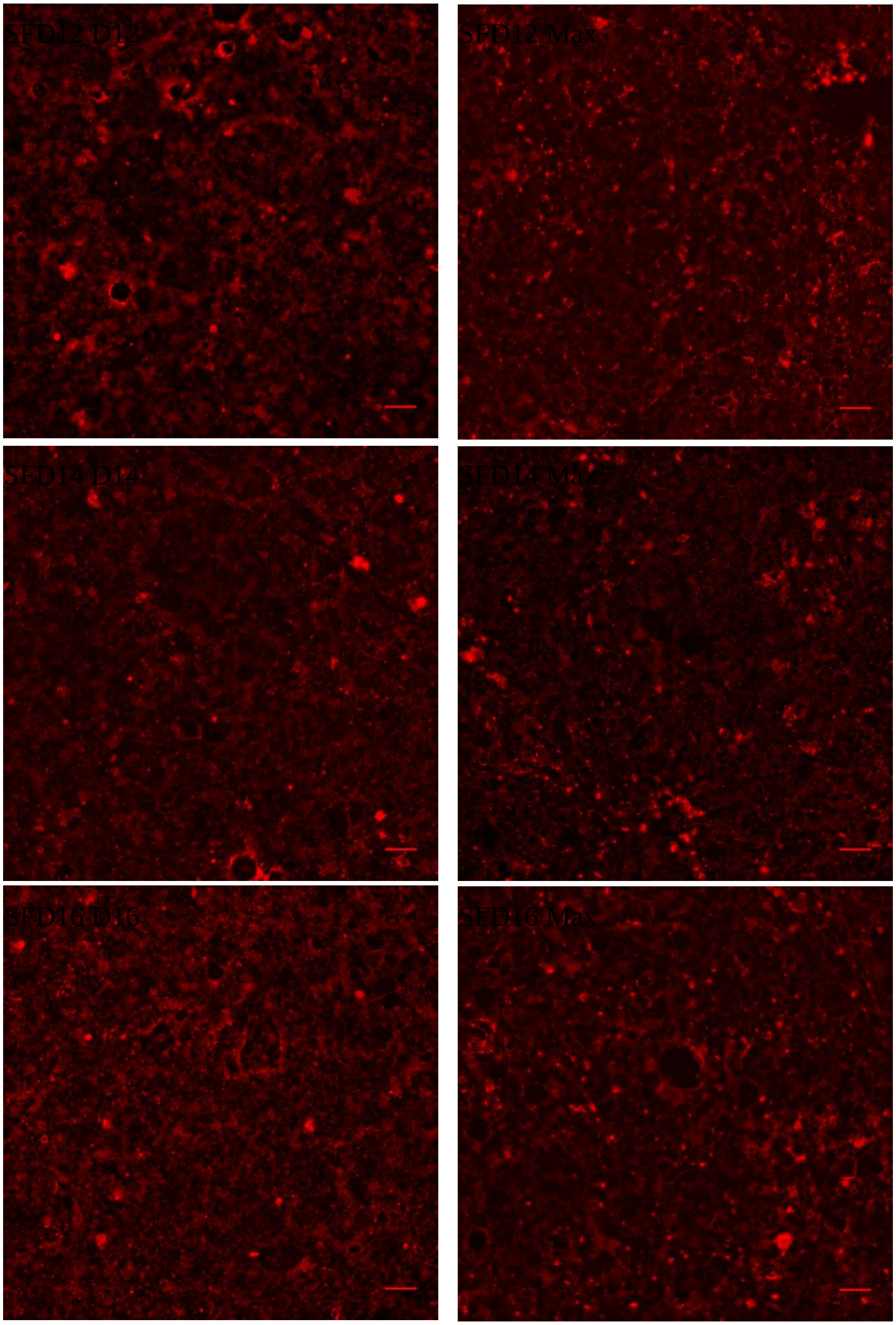
SFD12 D12
SFD12 Max
SFD14 D14
SFD14 Max
SFD16 D16
SFD16 Max

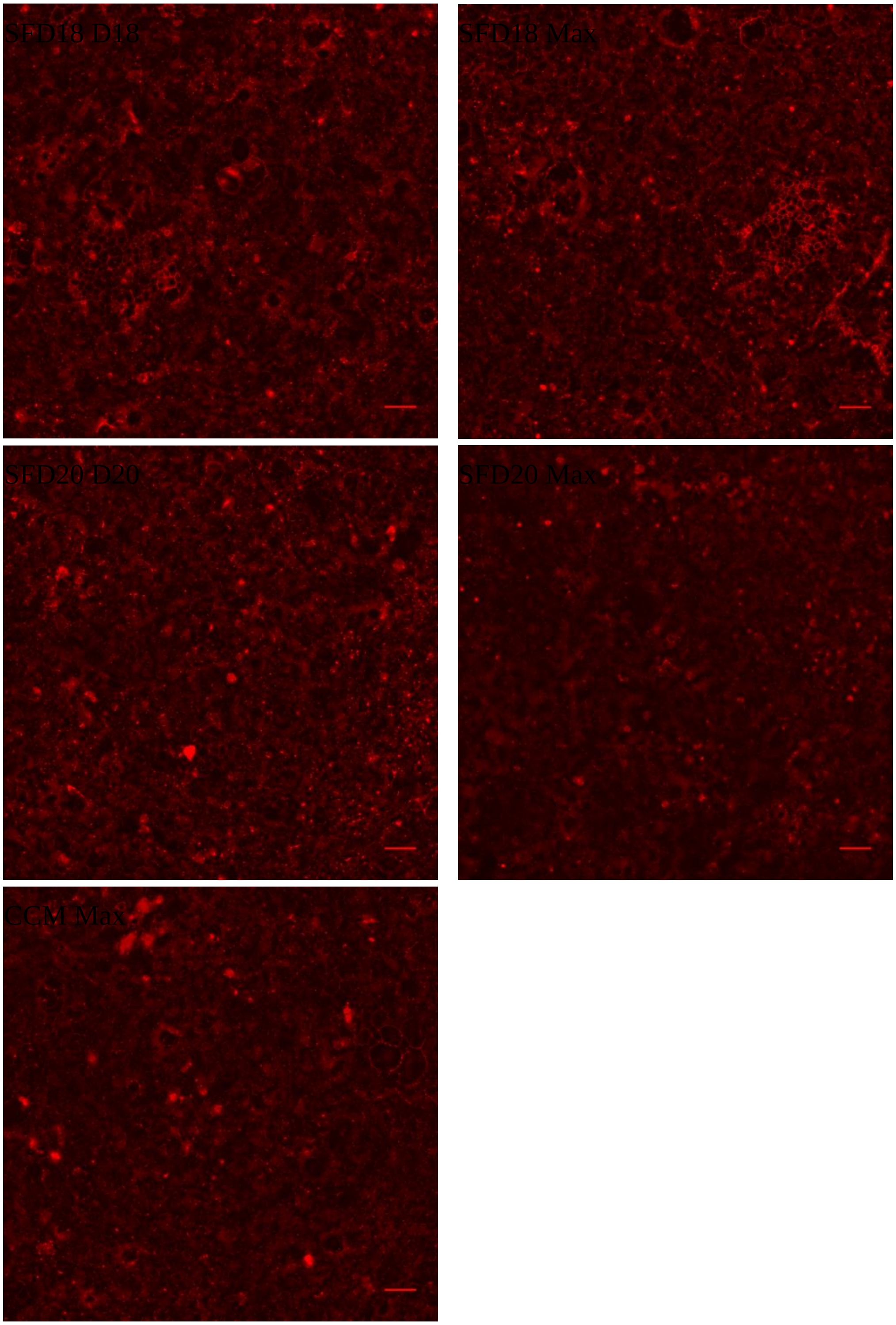

Figure S4: Representative images of ZO-1 staining in different culture conditions. Scale bar = 100 µm. SF = Serum free, D = day; D0 = cells changed to SF media on Day 0; SF D0 D0 = cells exchanged to SF media on day 0, fixed that day i.e. on day 0; SFD0 Max = cells exchanged to serum free media on day 0, fixed on the day these cultures achieve maximum resistance.

4.4 Occludin

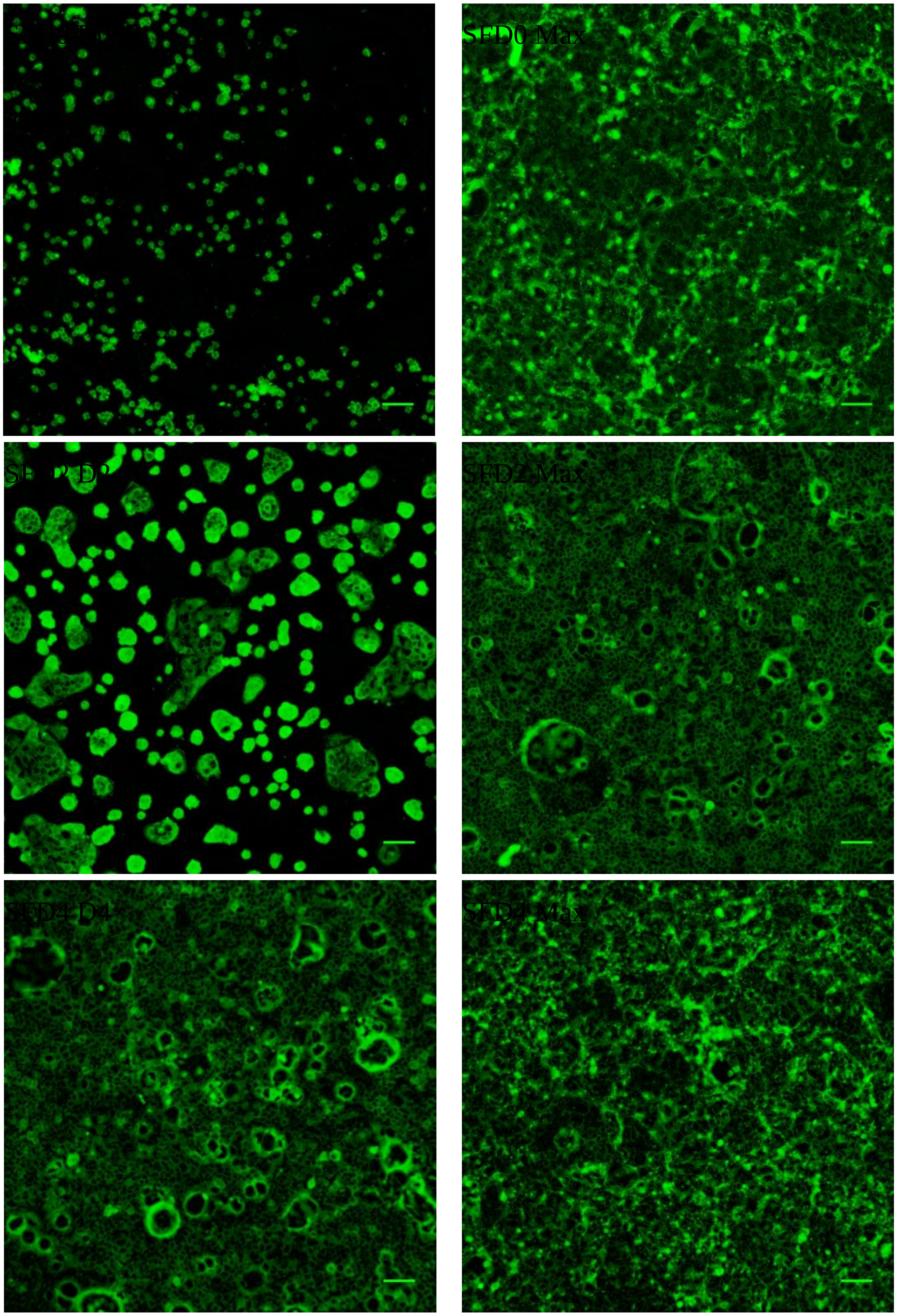

SFD0 Max
SFD2 Max
SFD4 D4
SFD4 Max

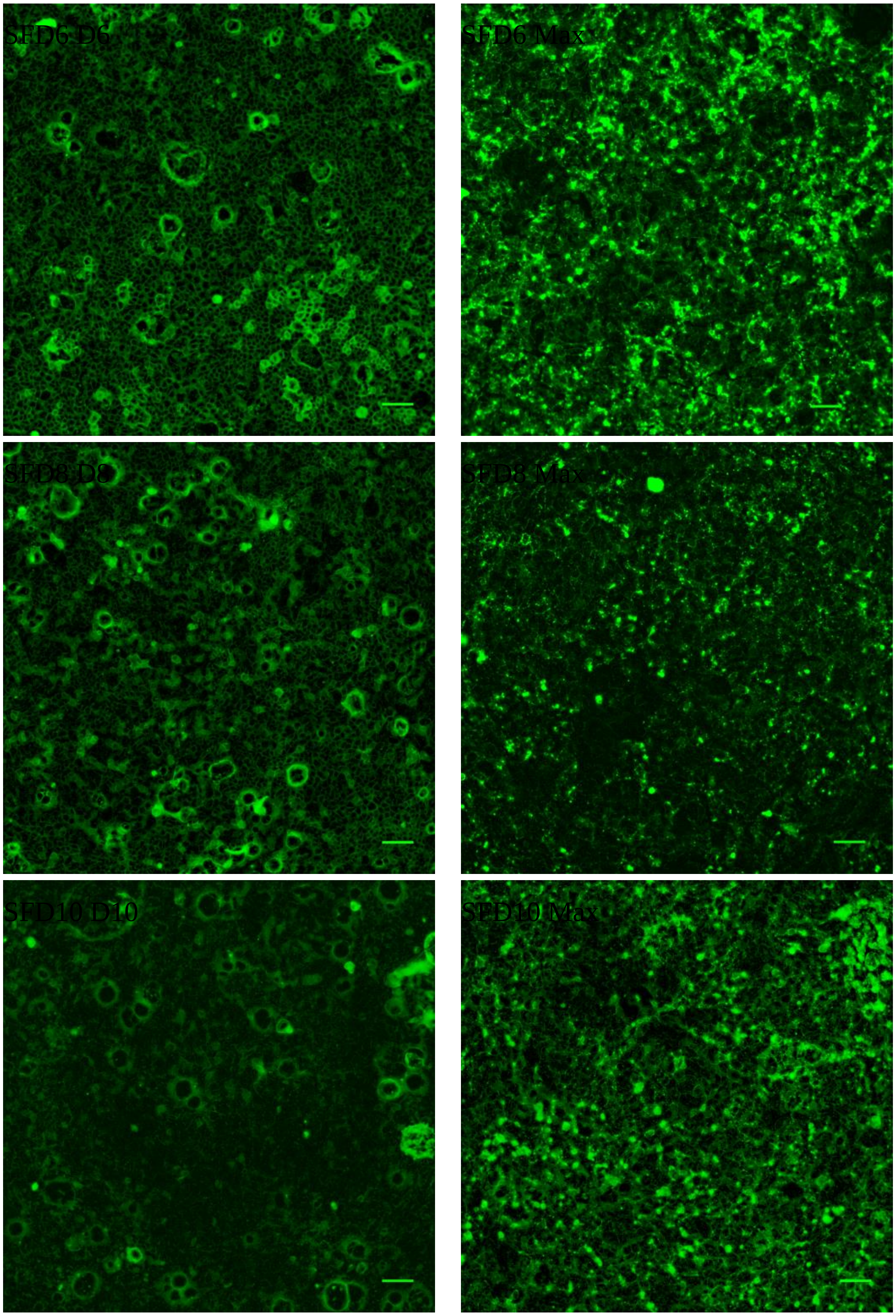
SFD6 D6
SFD6 Max
SFD8 D8
SFD8 Max
SFD10 D10
SFD10 Max

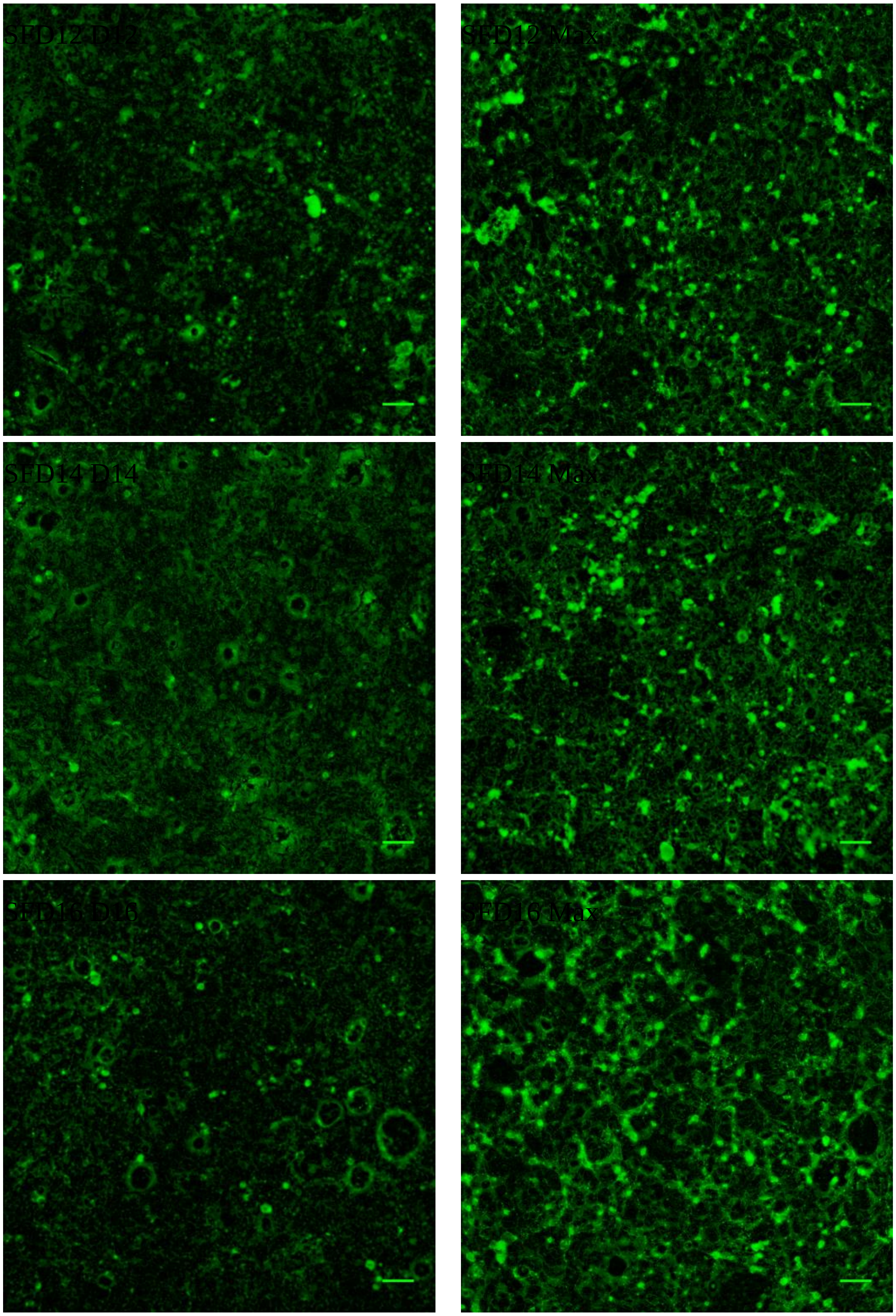

SFD12 D12
SFD12 Max
SFD14 D14
SFD14 Max
SFD16 D16
SFD16 Max

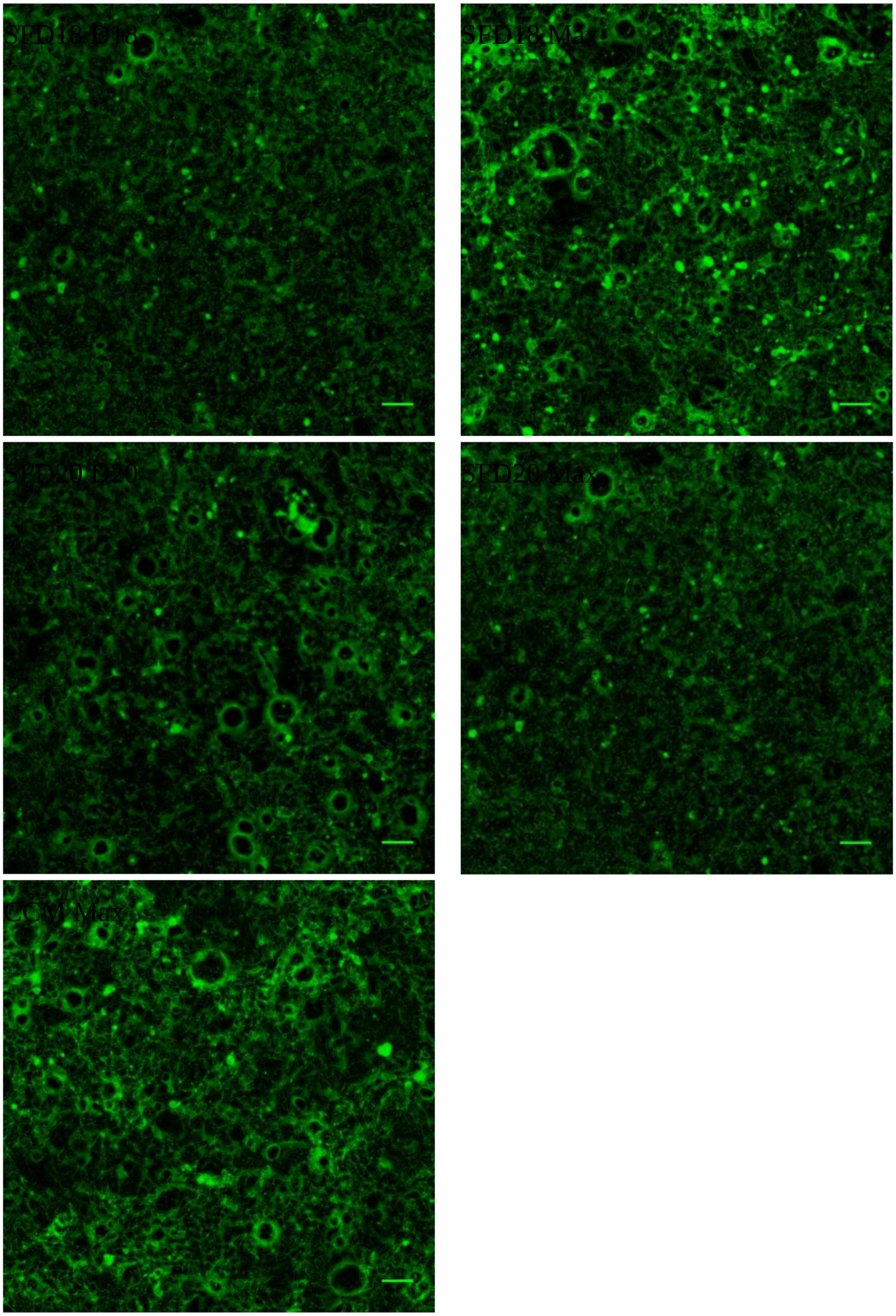

Figure S5: Representative images of occludin staining in different culture conditions. Scale bar = 100 µm. SF = Serum free, D = day; D0 = cells changed to SF media on Day 0; SF D0 D0 = cells exchanged to SF media on day 0, fixed that day i.e. on day 0; SFD0 Max = cells exchanged to serum free media on day 0, fixed on the day these cultures achieve maximum resistance.
